# Plant Roots and Phenology Drive the Spatio-Temporal Variability of Boreal Forest Floor Respiration

**DOI:** 10.3390/plants15040538

**Published:** 2026-02-09

**Authors:** Quan Zhou, Zonghua Wang, Meilian Chen

**Affiliations:** Fuzhou Institute of Oceanography, College of Materials and Chemical Engineering, Minjiang University, Fuzhou 350108, China; quanzoey@hotmail.com

**Keywords:** boreal forest, fine roots, metabolic continuum, mycorrhizal bridge, biological pulse, spatio-temporal variability, plant phenology, soil carbon dynamics

## Abstract

Understanding the drivers of soil carbon efflux is critical for predicting forest carbon cycles under climate change. This study investigates how plant roots and phenology govern the spatio-temporal variability of boreal forest floor respiration (Rf) in an ectomycorrhizal-dominated forest. By analyzing stabilized soil carbon fluxes (NEE, Ra, and Rh) one year after root exclusion in northern Sweden, we challenge the passive physicochemical paradigm. Results show that the spatial distribution and magnitude of Rf are primarily driven by plant roots, with Ra accounting for >60% of total efflux. The collapse of respiration in trenched plots confirms the mycorrhizal bridge as the essential conduit for these spatial patterns. Regarding temporal variability, we identified a biological pulse driven by plant phenology. After temperature-normalization, Ra maintained a strong seasonal peak in July and August. Notably, static drivers like fine root biomass failed to explain spatial variation (R < 0.3, *p* > 0.05), whereas dynamic NEE showed significant positive correlations (R = 0.52, *p* < 0.0001). This holistic perspective suggests that the forest floor operates as an integrated metabolic continuum, where root activity and phenological pump are the main regulating factors on carbon release. Future models should reposition plant–fungal phenology as the primary engine of soil metabolism.

## 1. Introduction

Boreal forests represent a critical global carbon (C) reservoir, characterized by an exceptional sensitivity to climatic shifts. Historically, the scientific community has approached forest floor respiration (Rf) through a reductionist framework, partitioning it into autotrophic (Ra) and heterotrophic (Rh) components under the assumption that their spatio-temporal variability is primarily dictated by passive environmental drivers, such as temperature, moisture, and litterfall availability [1,2,3,4]. While recent harmonized global assessments successfully predicted the mean respiratory intensity at broad scales using physicochemical factors, these static predictors frequently fail at the patch-level [4,5]. This suggests significant uncertainty stemming from localized environmental heterogeneity (e.g., micro-topography and nutrient distribution) and, perhaps more critically, the omission of active biological driving mechanisms regulated by host physiological states [6]. Recent modeling efforts have sought to address the vast spatial heterogeneity of soil respiration by employing “environmentally similar zoning” and integrating high-resolution vegetation productivity variables (e.g., GPP and EVI), which has substantially improved the predictive accuracy of global Rh estimates [7].

However, this passive paradigm overlooks the dominant role of plant roots and their associated fungi as active metabolic engines [8,9,10]. In boreal forests dominated by ectomycorrhizal (ECM) fungi, these organisms are not only superior in biomass and diversity but function as an essential extension of the root system, deeply regulating soil organic matter decomposition [11,12,13,14,15,16]. This symbiotic coupling establishes a highly integrated mycorrhizal bridge between plants and microbial networks. Operationally, this bridge is defined as an evolutionarily obligate biophysical infrastructure that extends from the Hartig net to the extraradical mycelium [17,18,19]. It functions as a dedicated metabolic conduit that transduces host photosynthetic energy into biochemical priming signals [20,21,22], thereby actively gating the decomposition and stabilization of soil organic matter.

This study challenges the traditional passive physicochemical paradigm, in which physical factors such as fine root production and litterfall fail to explain the observed spatial heterogeneity of respiration [5,17]. We propose that a phenological pump, governed by the allocation of photosynthates, orchestrates the seasonal rhythms of respiration across various temporal scales. By employing temperature-normalization techniques, we demonstrate that forest floor respiration maintains robust phenological signatures even after the thermodynamic effects are decoupled. This mechanism, driven synergistically by plant roots and phenology, reshapes the forest floor into a unified metabolic continuum. Operationally, this continuum is defined as a carbon flux system where plant-derived photosynthates act as the primary driver, physically and functionally coupling the host with the soil matrix through the root–ECM network. This system manifests as an emergent property of the forest holobiont, characterized by metabolic rhythms governed by host phenology rather than passive environmental thermodynamics [18,19,20,21,22,23,24]. Utilizing long-term trenching experiments in the Svartberget experimental forest, Sweden [25,26], we aim to verify the operational logic of this integrated living entity under steady-state conditions, providing a holistic perspective for predicting forest carbon dynamics.

The objectives of this study were: (1) to verify whether the boreal forest floor operates as a coupled living entity rather than a mere collection of independent decomposers; (2) investigate the underlying biological mechanisms of respiratory collapse following the severing of the mycorrhizal bridge; and (3) redefine and quantify the dominant role of photosynthetically-derived substrate flux and phenological rhythms in driving the spatio-temporal variability of forest floor respiration. Through this framework, we aim to provide an integrative perspective for predicting the evolution of forest carbon source/sink relationships under global warming.

## 2. Results

### 2.1. Dynamic Response of Forest Floor Respiration Components and Trenching Effects

#### 2.1.1. Seasonal Variation of Respiration

**Figure 1:**
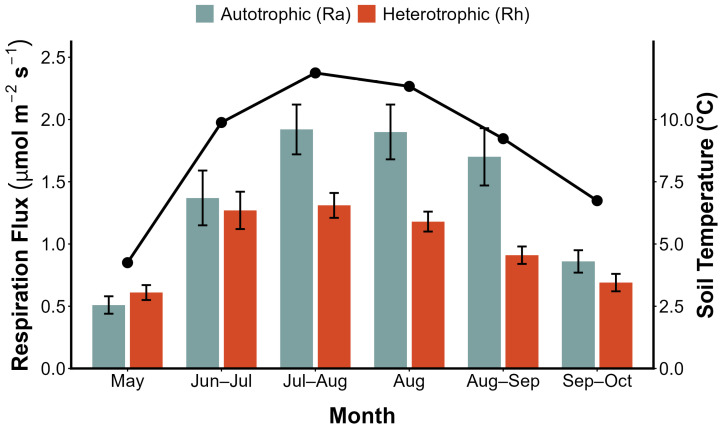
Dynamic response of forest floor respiration components (Ra and Rh) to seasonal fluctuations in soil temperature. The blue bars represent autotrophic respiration (Ra), the red bars represent heterotrophic respiration (Rh), and the black line indicates soil temperature at a depth of 5 cm. Experimental data were collected in 2017, one year after the trenching treatment, to ensure the system had reached a steady state and to eliminate the influence of initial dead root decomposition. The results show that Ra exhibited a significant seasonal biological pulse, with its peak highly synchronized with maximum soil temperatures and the peak plant growing season. In contrast, the fluctuations in Rh with temperature were smaller and more stable.

During observations in the 2017 growing season (May to October), the intact and trenched plots exhibited significant differences in flux (Figure 1). Trenching treatment led to a substantial decline in forest floor CO_2_ efflux, with heterotrophic respiration (Rh) contributing 29.9–39.1% to the total efflux. The Ra/Rh ratio ranged from 1.56 to 2.34, while autotrophic respiration (Ra) showed a distinct biological pulse, with its peak magnitude (reaching 1.92 μmol m^−2^ s^−1^) precisely synchronized with the maximum soil temperature and the peak of the plant growing season in July–August. In stark contrast, Rh remained at a low level (maximum 1.31 μmol m^−2^ s^−1^), representing a sluggish, passive biophysical baseline. This contrast provides strong evidence that Ra is not a simple passive physicochemical process, but rather an active metabolic flux driven by plant energy inputs.

#### 2.1.2. Steady-State Verification

**Figure 2:**
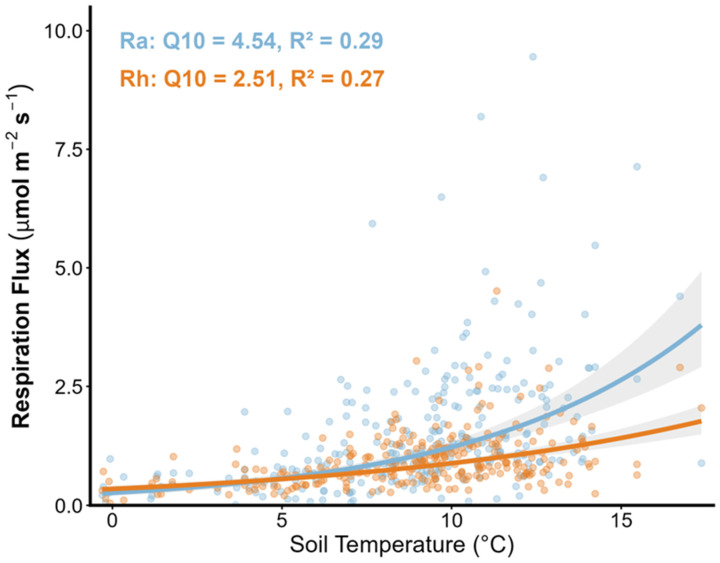
Temperature sensitivity of heterotrophic (Rh) and autotrophic (Ra) respiration in 2017. The relationship between soil CO_2_ efflux and soil temperature at a 5 cm depth was fitted using exponential regression models (R = a × ebT). After a one-year stabilization period following trenching, the Rh component (red dots and line) exhibited a consistent and predictable response to temperature fluctuations, with a calculated Q_10_ value of 2.51. The relatively low dispersion of Rh data points around the regression line suggests that the heterotrophic metabolic baseline had reached a steady state, effectively minimizing artifacts from residual root decomposition. In contrast, Ra (blue-green dots and line) showed a significantly higher temperature sensitivity (Q_10_ = 4.54) and greater variability, reflecting the additional influence of plant-driven carbon input pulses during the peak growing season.

After one year of physical isolation, the robust response of Rh to soil temperature in 2017 confirmed the steady state of the system (Figure 2). The temperature sensitivity of Rh (Q_10_ = 2.51) exhibited typical characteristics of microbial decomposition, with observations distributed uniformly around the fitted curve and no signs of pulses from dead root decomposition. In contrast, Ra showed extremely high temperature sensitivity (Q_10_ = 4.54), reflecting a strong surge of the plant energy engine during high-temperature periods. Additionally, there were no significant differences in soil temperature (at 5 cm and 10 cm depths) or soil moisture content between the control and trenched plots, ruling out the contribution of physical environmental changes to the decline in respiration.

#### 2.1.3. Temperature Normalization

**Figure 3:**
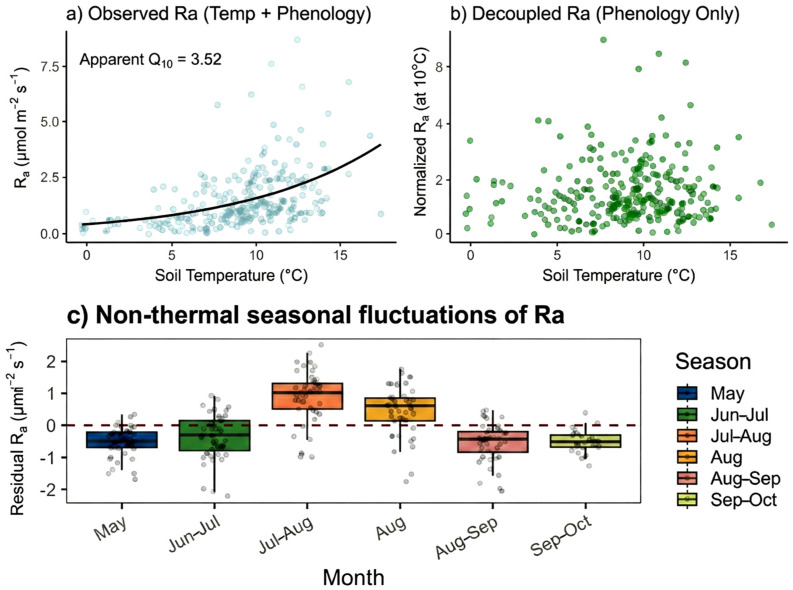
Deconvolution of temperature and phenological drivers of autotrophic respiration (Ra). (**a**) The apparent temperature sensitivity of observed Ra, showing a combined effect of metabolic kinetics and seasonal phenology (apparent Q_10_ = 3.52). (**b**) Temperature-normalized autotrophic respiration (R_a, norm_), revealing the decoupled metabolic intensity driven by phenology after removing the kinetic temperature effect using the site-specific Q_10_ function. (**c**) Thermal decoupling and non-thermal seasonal fluctuations of residual Ra. The boxplots demonstrate significant non-random variations after excluding thermal interference, with pronounced positive residuals during the peak growing season (July–August).

To isolate the biological drivers of autotrophic respiration, we performed a numerical deconvolution of the Ra flux to separate thermodynamic kinetics from seasonal phenological signals. The results show that the normalized Ra exhibited a significant seasonal peak in July–August rather than a stable baseline (Figure 3 and Figure S1). The observed Ra exhibited an inflated apparent temperature sensitivity (apparent Q_10_ = 3.52, Figure 3a), which represents a superimposed effect of Arrhenius-type kinetics and synchronized plant growth. However, upon decoupling these factors through temperature-normalization, the residual R_a, norm_ revealed a distinct metabolic intensity that operates independently of immediate thermal forcing (Figure 3b). The residual analysis provides compelling evidence for this mechanism: the non-random, significant variations in Ra residuals—characterized by pronounced positive deviations during July and August—confirm that the biological pulse occurs when the metabolic engine is at its peak investment phase (Figure 3c). These results demonstrate that the seasonal rhythm of Ra is not a passive reaction to warming but is fundamentally driven by a phenological pump of photosynthate allocation, which maintains high metabolic throughput even when temperature effects are mathematically excluded.

### 2.2. Temporal Dynamics and the Loss of the Biological Pulse

**Figure 4:**
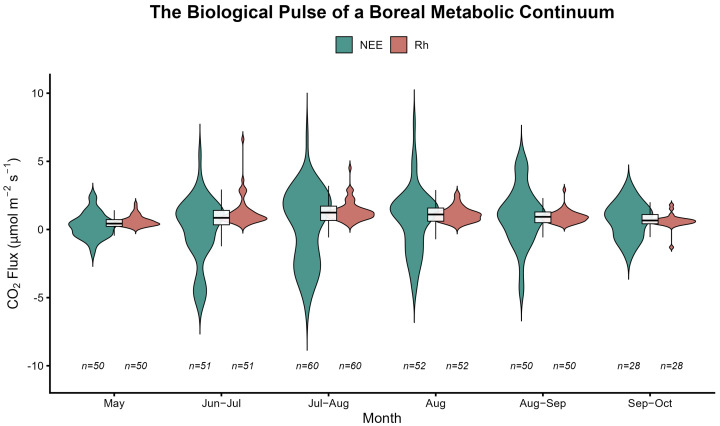
Seasonal synergy between net ecosystem exchange (NEE) and heterotrophic respiration (Rh). The violin plots illustrate the probability density and distribution of CO_2_ fluxes for NEE (green, representing the biological pulse of plant metabolism) and Rh (red, representing the decoupled soil baseline) from May to October 2017. Within the violin plots, the white boxes indicate the interquartile range (IQR); the central horizontal line represents the median; and the whiskers extend to the most extreme data points within 1.5 times the IQR. While NEE exhibited high-amplitude fluctuations and distinct seasonal pulses—peaking during the peak growing season (July–August)—the Rh component remained remarkably stable and constrained. We observed a complete disappearance of the biological pulse in trenched plots (Rh). The increased sample size in July–August (*n* = 60) is attributed to replicate measurements taken during equipment calibration. Conversely, data collection in late Sep–Oct (*n* = 28) was curtailed by early snow cover on the forest floor.

By comparing the seasonal fluctuations of NEE and Rh, we observed a functional transition of the system from coupled to decoupled (Figure 4).

Analysis indicates that the Net Ecosystem Exchange (NEE, green) exhibits significant high-amplitude fluctuations and distinct seasonal pulse characteristics. During the peak growing season (July–August), the distribution of NEE was most expansive, with the most pronounced peaks, reflecting metabolic activity driven by plant energy input.

In sharp contrast, the distribution of heterotrophic respiration (Rh, red) was highly constrained and remained remarkably stable. Despite drastic seasonal variations in external environmental conditions (e.g., canopy photosynthetic intensity), the Rh component—representing baseline soil emissions—did not fluctuate commensurately. This “decoupling” phenomenon suggests that when the direct contribution of plant roots is excluded (via trenching treatments), carbon release derived solely from the microbial decomposition of organic matter maintains a relatively constant baseline, remaining less susceptible to instantaneous environmental perturbations.

The central scientific finding illustrated here is the complete disappearance of biological pulses within the trenched plots (Rh). Although the surrounding canopy maintains high-intensity photosynthetic metabolic activity, the physical isolation imposed by trenching severs the transport of photosynthates to the rhizosphere, resulting in the loss of seasonal pulsing in forest floor carbon release. Therefore, forest floor carbon release is not merely a passive physiochemical process driven by ambient temperature and moisture; rather, it is a continuous metabolic process dominantly governed by plant-derived energy flux.

### 2.3. Plant Biomass as a Driver of Metabolic Intensity

**Figure 5:**
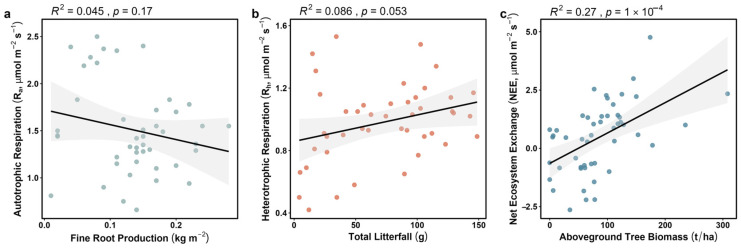
Decoupling of spatial drivers for respiration components. (**a**) Linear regression between autotrophic respiration (Ra) and fine root production across 50 plots. Solid lines represent non-significant linear fits (R^2^ = 0.045, *p* = 0.17). (**b**) Linear regression between heterotrophic respiration (Rh) and total litterfall input. Solid lines represent non-significant relationship (R^2^ = 0.086, *p* = 0.053). (**c**) Significant relationship between mean Net Ecosystem Exchange (NEE) and Aboveground Tree Biomass (AGB) across the study plots (R^2^ = 0.27, *p* < 0.001) with 95% confidence intervals. The strong positive correlation suggests that stand structure significantly influences the magnitude of net ecosystem exchange.

Figure 5 demonstrates a spatial asymmetry in forest carbon cycling processes. While subdivided respiration metrics (Ra and Rh) exhibited a spatial decoupling from direct biomass inputs, the net ecosystem exchange (NEE) remained highly dependent on stand succession stage. This finding emphasizes that when constructing spatial models of forest carbon balance, local respiratory components should not be simplified as mere inputs from the static biogenic substrate pool. Instead, greater attention must be directed toward the regulatory role of succession-driven photosynthetic carbon input capacity on total ecosystem fluxes.

Ra vs. Fine Root Production (Figure 5a): The correlation coefficient R = −0.21 with *p* = 0.17. While *p* > 0.05 suggests that these specific environmental factors exerted little detectable influence on respiration variance within our experimental context, instead, these fluctuations are primarily regulated by dynamic physiological processes, supporting the role of high-throughput energy flux from roots and associated fungi as the dominant driver of belowground carbon dynamics.

Rh vs. Total Litterfall (Figure 5b): The correlation coefficient R = 0.29 with *p* = 0.053, highlighting the limitations of litter input. Although the *p*-value was close to the significance threshold of 0.05, it was considered only marginally significant. This marginal significance (*p* = 0.053) suggests that aboveground litterfall provides limited explanatory power for Rh variance in this system. We acknowledge that our analysis did not explicitly control for gradients in stand age or soil fertility; however, given our extensive sampling across diverse forest conditions, the lack of a robust correlation despite these underlying variations suggests that litterfall is not the primary driver. This aligns with the findings of Clemmensen [8], which indicate that as boreal forests age, organic matter accumulation is increasingly dominated by the long-term sequestration of roots and fungal necromass rather than aboveground litter.

NEE vs. Aboveground Tree Biomass (AGB) (Figure 5c): Net ecosystem exchange (NEE) showed a significant positive correlation with the total AGB (R = 0.52, *p* < 0.001). In contrast to the poor performance of single biogenic substrates (such as roots or leaves alone), the biomass scale of the entire system demonstrated high explanatory capability. This strong correlation indicates that forest stand structure—represented here by Aboveground Biomass (AGB)—serves as the core spatial driver determining the capacity for Net Ecosystem Exchange (NEE).

## 3. Discussion

### 3.1. The Metabolic Essence of Respiration Collapse

The most striking observation of this study was the dramatic collapse of forest floor respiration during the steady-state phase (one year post-trenching) following the cessation of carbon supply via the extraradical mycelium (ERM). This phenomenon suggests that while trenching achieves the physical separation of components, it induces a profound disruption of energy continuity within the plant–soil system. This functional decoupling not only cuts off the physical supply of carbon but, more critically, severs the mycorrhizal bridge that maintains the metabolic rhythm of the forest floor. Accordingly, we propose that boreal forest floor respiration should not be viewed as a simple summation of independent carbon pools, but as an integrated metabolic continuum driven by real-time energy [27,28,29].

Our monitoring data provide direct evidence for this functional fracture: during the steady-state phase, a fundamental decoupling occurs between soil respiration and its physical substrates. Despite the biogenic substrate accumulation of static litter and necromass, the heterotrophic respiration flux (Rh) remained at a low and passive level with minimal spatial heterogeneity (R^2^ = 0.29, *p* > 0.05; Figure 5b). This finding strongly challenges the traditional passive physicochemical paradigm and aligns with the perspectives of peer researchers—specifically, that in the absence of a functional mycorrhizal bridge, the mere physical presence of soil organic matter does not guarantee metabolic activity [8,14,16].

Further quantitative analysis revealed that the high Ra/Rh ratio (1.56 to 2.34, Figure 1) in this boreal forest indicates that the majority of carbon release is a direct manifestation of plant–fungal symbiotic metabolism. The biological pulse observed in intact plots is primarily driven by plant-derived energy flux rather than the spontaneous decomposition of soil organic matter. This mechanism is robustly corroborated by isotope tracing studies; for example, previous studies have demonstrated that dominant boreal species prioritize the allocation of photoassimilates for aboveground growth in the early growing season (June), while the proportion allocated belowground increases substantially in July and August (reaching 32–44% and 12–24%, respectively) [30]. This seasonal “transition of belowground investment” provides a direct mechanistic explanation for the late-summer metabolic peak observed in this study, confirming that the rhythm of forest floor respiration is fundamentally a lagged response to the phenological allocation of plant photosynthates.

Once the mycorrhizal bridge is severed, the system undergoes a functional metabolic dormancy, transitioning from an active metabolic continuum to a sluggish, passive system driven solely by temperature. Notably, the disappearance of the long tail (high-flux hotspots) in the spatial distribution of Rh (Figure 4) provides conclusive evidence that without the injection of real-time energy flux from plants, soil microbial communities lose the autonomous capacity to generate high-intensity metabolic hotspots. This shift from life-driven to a physical baseline reveals the absolute dependence of the boreal forest floor carbon cycle on continuous plant energy flux [27,28,29].

### 3.2. Phenology-Driven Rhythm vs. Failure of Physical Drivers

This study found that the temperature sensitivity of autotrophic respiration (Ra, Q_10_ = 4.54, decoupled Q_10_ = 3.53) was significantly higher than that of heterotrophic respiration (Rh, Q_10_ = 2.51) (Figure 2). This substantial response bias provides decisive evidence that plant input serves as the core engine of subsurface metabolism. The Rh contribution (29.9–39.1%) and the dominance of Ra (>60%) following trenching indicate that in this boreal forest ecosystem, the plant-driven autotrophic component modulates the overall rhythm of forest floor carbon release (Figure 1). After removing the thermodynamic effects of temperature, the normalized Ra maintained a strong seasonal peak in July and August (Figure 3). This phenological pulse, independent of the temperature background, conclusively demonstrates that the pulse of forest floor respiration is essentially driven by the phenological pump of plant photosynthates [30,31,32]. This plant-mediated biological pulse infuses the forest floor with activity exceeding the physical baselines through instantaneous energy flow. Once this vital connection is severed, the system inevitably reverts to a mediocre and sluggish state governed purely by physical thermodynamics due to the loss of the priming and control effects of root-derived carbon on microbial metabolism [15,17].

Notably, this study challenges the existing passive physicochemical paradigm that primarily attributes carbon release to abiotic thermodynamic cues (e.g., temperature kinetics) and the decomposition of static biogenic substrates. While temperature drives temporal variation, our normalized data confirm its inability to account for the spatial heterogeneity and canopy-synchronized phenological pulses observed in this system. Traditional static biogenic substrates, such as fine root biomass and aboveground litterfall, show low explanatory capability for the spatial variation of respiration compared to the dynamic influence of the phenological pump (Figure 3). The observed disappearance of seasonal pulses in Rh after trenching indicates that metabolic intensity is not determined by the size of static substrate pools, but by the intensity of the energy flux delivered via the extraradical mycelium (ERM). In contrast, the significant positive correlation between net ecosystem exchange (NEE) and total aboveground biomass (AGB) further confirms the existence of a metabolic continuum. Forests with higher AGB possess a more robust phenological pump—an active electron flow channeled underground via the mycorrhizal bridge—thereby driving overall forest floor metabolic levels. This is highly consistent with the conclusions of other empirical studies regarding the absolute dominance of fungi in boreal forests [12,13,14,33], where fungi, as an extension of plant life, dominate the turnover processes of soil organic matter.

Our observation that static biogenic factors fail to explain spatial variability while dynamic proxies like NEE do is highly consistent with recent global data-driven assessments. For instance, a recent study demonstrated that vegetation productivity variables (e.g., GPP and EVI) exhibit the highest importance in predicting Rh across diverse environmental zones [7], reinforcing the theory that vegetation serves as the primary engine for subsurface metabolic activity by providing real-time carbon substrates. This framework is conceptually paralleled by emerging evidence that trace gas fluxes on tree bark are similarly modulated by the host’s physiological state and nutrient exudation [34]. Such isomorphism underscores that the active biological governance of ecosystem fluxes—extending from the rhizosphere to the canopy—represents a unified regulatory system driven by the host’s metabolic energy distribution.

### 3.3. The Critical Role of Root-Associated Fungi

As demonstrated in Section 3.1, the functional decoupling between Rh and litter pools suggests that the stability of the belowground carbon pool is independent of biogenic substrate quantity. In the second year following trenching, Rh remained at an extremely low level and was relatively unresponsive to temperature, despite the presence of significant residual root and fungal necromass. This observation strongly validates the high biochemical recalcitrance of fungal remains in boreal forests [8]. Such stability implies that in the absence of the priming effect triggered by fresh plant carbon sources, the fungal necromass accumulated over long periods is difficult for purely saprotrophic microbes to degrade [14,35].

These findings are highly consistent with the conclusions drawn in previous studies [8,14,16]. The security of the subsurface carbon pool in boreal forests depends not only on environmental temperature but also on the integrity of the metabolic coupling between plants and fungi. The increasing thickness of the organic layer in boreal forests is not due to the simple accumulation of aboveground litter, but relies on the continuous bottom-up accretion of roots and fungal remains at deeper layers [12,17]. The biochemical inertness of this subsurface carbon pool explains why, even under warming scenarios, microbes cannot effectively decompose these accumulated residues once the plant–fungal continuum is broken. Therefore, our study functionally demonstrates that subsurface carbon sequestration in boreal forests is essentially a functional decoupling resulting from the interruption of energy flux, which carries more systemic significance than traditional physicochemical explanations. Although this study did not directly measure the chemical composition of the residues, the complete disappearance of respiration pulses post-trenching (Figure 4), combined with the low sensitivity of Rh to litter input (Figure 5b), functionally confirms the biochemical inertness of the boreal forest subsurface carbon pool. The identified root–fungal metabolic continuum conceptually resonates with emerging evidence that plant-associated microorganisms—across diverse interfaces including tree bark—actively modulate ecosystem–atmosphere exchange. Such isomorphism suggests a unified regulatory framework for host-mediated gas fluxes throughout the forest profile [34].

**Figure 6:**
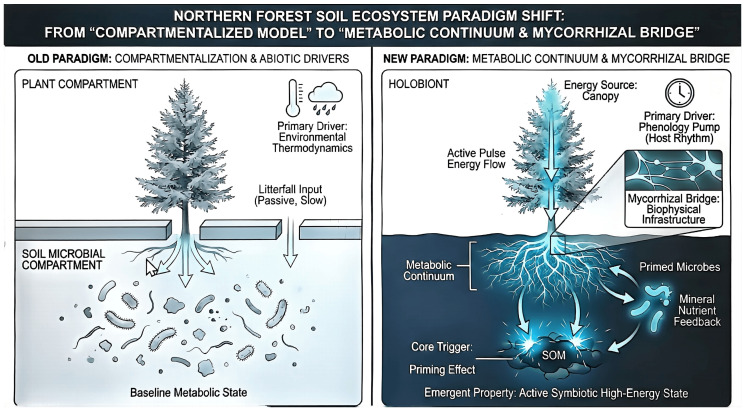
Conceptual model of the forest soil ecosystem paradigm shift. Comparison between the traditional compartmentalized model driven by environmental thermodynamics (left) and the proposed metabolic continuum (right). The mycorrhizal bridge serves as the biophysical infrastructure that synchronizes canopy energy pulses with soil microbial activity via the phenology pump. This symbiotic coupling triggers the rhizosphere priming effect, transitioning the forest floor from a baseline metabolic state to an active, high-energy symbiotic state. Such biological governance transcends compartmental boundaries, identifying the forest floor as a single metabolic continuum.

To encapsulate the transition from a passive pool-based view to an active metabolic continuum, we propose a conceptual model (Figure 6) that highlights the mycorrhizal bridge as the core biophysical infrastructure. This framework integrates the phenology pump and the metabolic continuum, illustrating how host-mediated energy pulses drive the ecosystem into an active symbiotic high-energy state [18,19,20,21,22,23,24,35].

## 4. Materials and Methods

### 4.1. Study Region

This study was conducted in the Krycklan Catchment (64°14′ N, 19°46′ E), a forest research site of the Swedish University of Agricultural Sciences [25,26,36]. The Krycklan Catchment is located close to the ICOS-Svartberget field station, approximately 50 km northwest of Umeå in northern Sweden. The climate is a cold temperate humid type with persistent snow cover during the winter season that usually lasts from November to March. The 30-year (1981–2010) mean annual temperature is 1.8 °C. The annual precipitation is 614 mm [25,36,37].

The catchment is predominantly forested (87% coverage), and comprised mainly of second-growth stands. The dominant tree species are Scots pine (*Pinus sylvestris* L.) (63%) and Norway spruce (*Picea abies*) (26%). Deciduous species like Birch (*Betula* sp.), Trembling aspen (*Populus tremula*), and Ash (*Fraxinus excelsior*) are also present in many stands throughout the catchment. The forest understory layer is dominated by ericaceous dwarf shrubs including lingonberry (*Vaccinium vitis-idaea*), bilberry (*Vaccinium myrtillus*), and black crowberry (*Empetrum hermaphroditum*) [25,38].

### 4.2. Experimental Design

**Figure 7:**
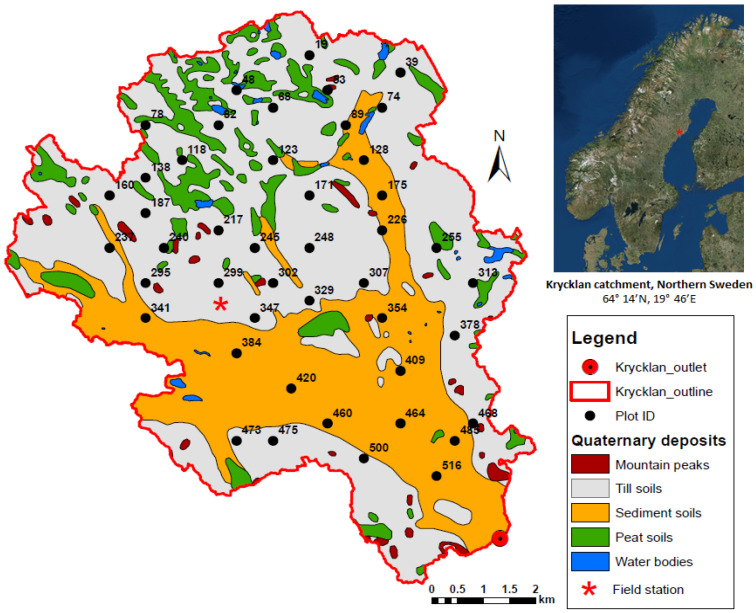
Simplified soil types and selected plots (50) across the Krycklan catchment.

A total of 50 study plots were selected from a grid of 550 potential plots across the Krycklan catchment (Figure 7). The selection criteria ensured an even distribution regarding geological locations, soil types, and forest stand biomass.

In 2016, a total of 50 plots were selected out of a grid of 550 plots (300 × 300 m) across the Krycklan catchment. In each plot, two permanent measurement collars were established: Control—Used for measuring Net Ecosystem Exchange (NEE) and total Forest Floor Respiration (Rf); and (2) Trenched—Established where trenching and vegetation removal were applied to exclude root respiration, allowing for monitoring of Heterotrophic Respiration (Rh). Eliminating Artifacts: In the early stages of trenching, the decomposition of dead roots causes a peak in respiration, which can mask the true collapse mechanism. Therefore, we measured the forest floor respiration carbon flux one year after trenching (spring 2017), when the dead root decomposition had stabilized and the physicochemical properties of the remaining organic matter had reached a new steady state. Synchronicity of Control Plots: Ensuring that the microclimate (temperature and moisture) of the intact plots remained highly similar to that of the trenched plots.

Vegetation characteristics were surveyed within inventory subplots centered on each flux collar. Three litter traps (R = 0.25 m) were installed 10 m north of each collar to quantify litter production, which was collected and sorted into the hardwood ratio and coniferous ratio.

### 4.3. In Situ Soil CO_2_ Efflux

**Figure 8:**
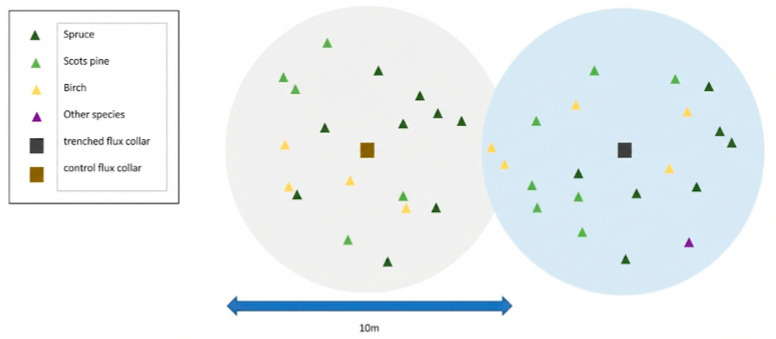
Spatial distribution of vegetation and flux collars within the experimental plots. The gray and blue circular areas represent the control and trenched plots, respectively. The blue double-headed arrow indicates a spatial scale of 10 m. Colored triangles denote the specific locations of Spruce, Scots pine, Birch, and other tree species relative to the central flux collars (squares) for the Control and Trenched treatments at a 10 m spatial scale.

CO_2_ fluxes were measured using a static chamber system consisting of permanent aluminum collars and a removable chamber (45 × 45 × 15 cm) (Figure 8). Measurements were conducted over six periods during the 2017 growing season, ranging from May to October.

CO_2_ flux was analyzed using two devices: an LGR Greenhouse Gas Analyzer (Model GGA-24EP, ABB Inc., San Jose, CA, USA) for Acts as the primary measurement unit to precisely capture real-time dynamic changes in CO_2_ fluxes and a Vaisala Handheld CO_2_ Meter (Model MI70, Vaisala Oyj, Vantaa, Finland) for ambient CO_2_ concentrations. Cross-validation showed no significant difference between analyzers (R^2^ = 0.927).

The measurement protocol proceeded as follows:

NEE: Measured at control collars using a transparent chamber.

Rf and Rh: Measured at the control and trenched collars, respectively, by covering the transparent chamber with an black tarp to exclude light and inhibit photosynthesis.

Duration: Each measurement involved a 3-min chamber closure following ambient air flushing.

### 4.4. Environmental Monitoring

Biotic and abiotic factors were monitored concurrently with flux measurements or obtained from associated studies.

Light Availability: Photosynthetic Photon Flux Density (PPFD) was measured using a PAR sensor, held 30 cm above the forest floor during NEE measurements.

Microclimate: Soil moisture content was measured using a GS3 Ruggedized Sensor (Decagon Devices, Pullman, WA, USA). Soil temperature was recorded at 5 cm and 10 cm depths using a handheld probe, and air temperature was measured at a 1.3 m height.

Stand structure: Previously known data included in this study were soil type, humus layer depth, management practice, aboveground stand biomass, and dominant tree species. Data for Leaf Area Index (LAI) were sampled using LICOR-LAI2200C (LI-COR Biosciences, Lincoln, NE, USA).

Roots and litter: Fine root production was measured using soil coring techniques, Belowground biomass in terms of fine roots (<2 mm diameter) was measured via sequential coring in the early season (June 2017). Three litter traps (R = 0.25 m) were installed 10 m north of each collar to quantify litter production. Litter was collected monthly from May to October, then transported to the laboratory. The collected litter samples were oven-dried at 65 °C to a constant weight (minimum 48 h) using a forced-air oven. After drying, the samples were weighed immediately to the nearest 0.01 g to determine the dry matter content.

### 4.5. Statistical Analysis

We utilized multiple regression analysis (MRA) to disentangle the drivers. Spatial drivers were analyzed using seasonal means (*n* = 50), while temporal drivers were analyzed using the full dataset. All statistical analyses were performed in R (Version 4.5.2, https://www.r-project.org/) and Python (Version 3.1.4, https://www.python.org/).

## 5. Conclusions

Through a long-term trenching experiment in a boreal forest of northern Sweden, this study challenges the traditional reductionist paradigm, proposing instead an integrated metabolic continuum driven by plant roots (with associated fungi) and phenology.

First, the experimental data revealed a dramatic collapse in soil respiration one year after the cessation of root inputs. The real-time energy flux served as the core engine driving metabolic activity on the forest floor. Second, traditional physical drivers, such as fine root productivity and aboveground litterfall, showed extremely low explanatory power for the spatial variation of respiration. In contrast, net ecosystem exchange (NEE) and total aboveground biomass exhibited significant correlations. Finally, the trenching data demonstrated that once deprived of the metabolic support from plant photosynthates, existing microbial networks rapidly enter a state of dormancy or decline, while residual fungal necromass in the soil exhibits high biochemical recalcitrance.

In conclusion, our study identified the mycorrhizal bridge as the essential biophysical infrastructure that transitions the forest floor from a passive substrate pool into an active metabolic continuum. This paradigm shift demonstrates that forest carbon sinks are not merely regulated by abiotic thermodynamics, but are actively governed by the phenology pump of the host tree.

While our results clarify the physiological governance of soil respiration, this study has limitations that merit acknowledgment. First, the identification of the mycorrhizal bridge relies on respiratory flux dynamics rather than the direct quantification of fungal mycelial biomass or turnover rates. Second, our observations are restricted to a single annual cycle, which may not fully capture the inter-annual variability of carbon allocation typical of late-successional boreal forests. Finally, the absence of biochemical analysis for fungal necromass precludes a precise tracing of the molecular stabilization of root-derived inputs into long-term carbon pools. Addressing these gaps will further refine our understanding of the belowground-driven sequestration model.

## Data Availability

The original contributions presented in this study are included in the article. Further inquiries can be directed to the corresponding authors. Data and the R-based analysis scripts used in this study are available from the corresponding authors upon request.

## Supplementary Materials

The following supporting information can be downloaded at: https://www.mdpi.com/article/10.3390/plants15040538/s1, Figure S1: Model performance and sensitivity analysis. The sensitivity analysis (Figure S1a) using bootstrapping (n = 1000) demonstrated a robust and stable estimation of the apparent Q_10_ (mean ≈ 3.75). The normal distribution of the bootstrapped values confirms that our model is not overly sensitive to individual outliers.
