# Supplementary material for: Plant Roots and Phenology Drive the Spatio-Temporal Variability of Boreal Forest Floor Respiration
Source: Plants (Basel). 2026 Feb 9;15(4):538. doi: 10.3390/plants15040538 (PMC12944008; doi:10.3390/plants15040538)
Supplement: Supplementary file 1 [file plants-15-00538-s001.zip › plants-4136102-supplementary.pdf]

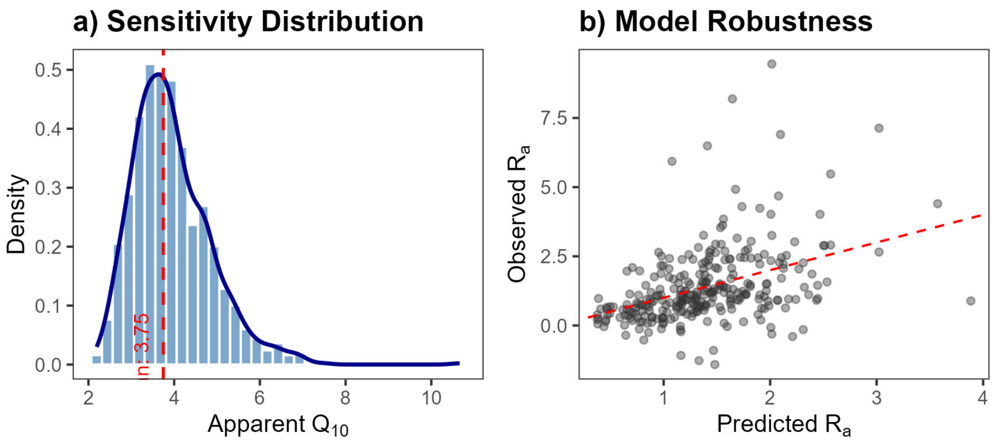

Figure S1. Model performance and sensitivity Analysis. (a) The sensitivity analysis using bootstrapping (n = 1000) demonstrates a robust and stable estimation of the apparent Q_{10} (mean $\approx$ 3.75). The normal distribution of the bootstrapped values confirms that our model is not overly sensitive to individual outliers. (b) Observed vs. predicted Ra values. The 1:1 dashed line demonstrates the reliability of the exponential model as a baseline for the phenological decoupling shown in Figure 3.
